# Pulsed field ablation in common inferior pulmonary trunk

**DOI:** 10.1007/s10840-022-01412-9

**Published:** 2022-11-09

**Authors:** Ashish Mittal, Noel Fitzpatrick, Gabor Szeplaki

**Affiliations:** 1 Atrial Fibrillation Institute, Mater Private Hospital, 71 Eccles Street, Dublin 7, D07 T92C Ireland; 2 Royal College of Surgeons in Ireland, Dublin, Ireland

**Fig. 1:**
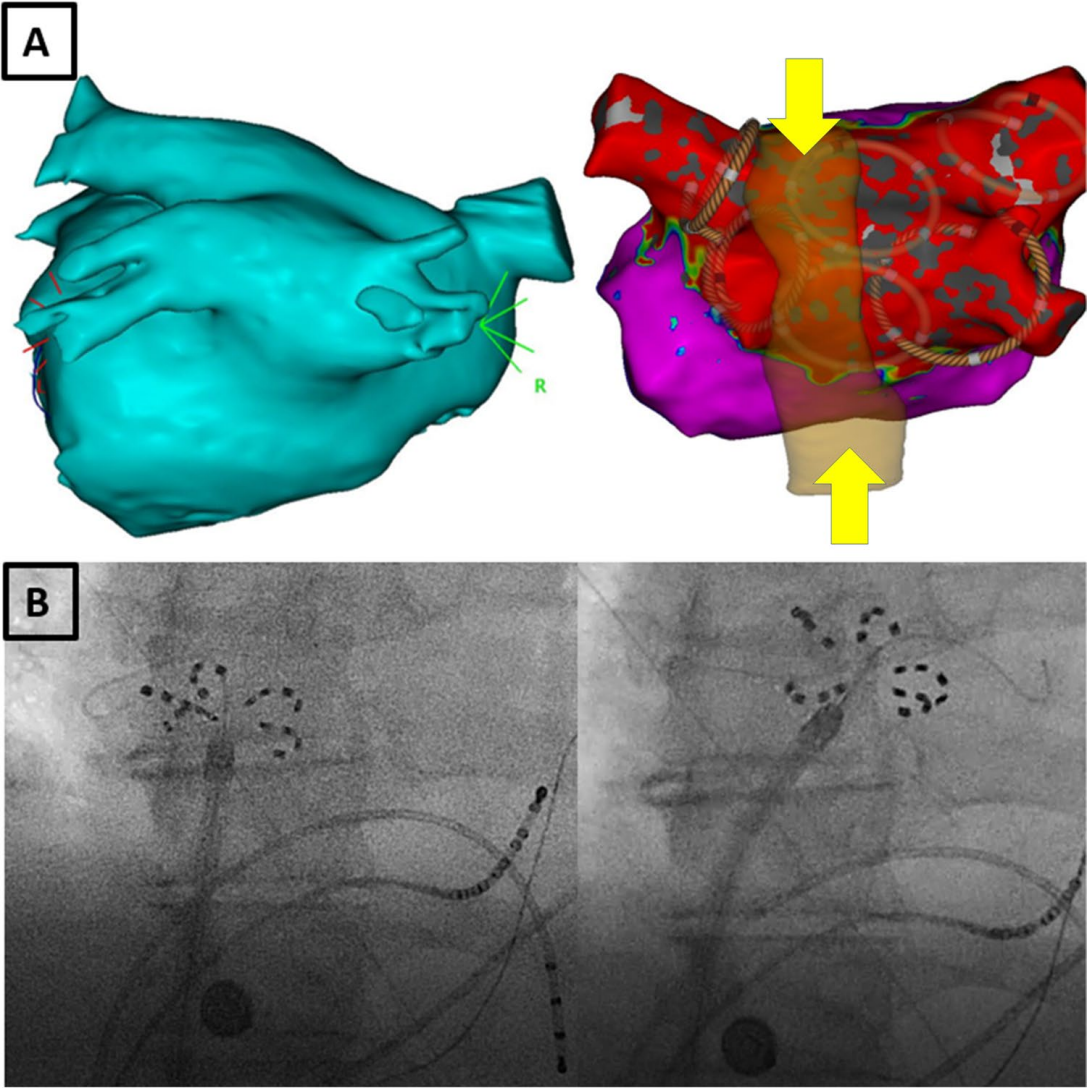
**A** Left: Computer tomography generated segmentation of the left atrium illustrating the common inferior trunk in a posterior-anterior (PA) view. When comparing the CT generated segmentation with the EAM map, note the subtly of this anatomical variant when relying on the EAM map alone, emphasizing the importance of performing pre-procedure imaging. Right: Post ablation bipolar voltage map of the LA with projected positions of the FaraWave catheter across LA (brown circular catheter) to isolate the pulmonary veins and posterior wall. Also note the CT generated segmentation of the esophagus (arrows) demonstrating its intimate relationship with the common trunk. **B** Fluoroscopic images of the FaraWave catheter positioned in the common inferior trunk with J wire in right branch (left) and in left branch of CIPT. One can easily visualize based on these fluoroscopic images the heavy thermal insult the esophagus would sustain if a typical point-by-point ablation radiofrequency ablation technique was used

A 65-year-old male patient with symptomatic drug refractory persistent atrial fibrillation elected to undergo pulmonary vein isolation after a failed cardioversion attempt (CHA_2_DS_2_-VASc 1, mEHRA2a). Pre procedure computed tomography of the left atrium (CTLA) confirmed the presence of a common inferior pulmonary trunk (CIPT; Fig. 1A, left image).

The procedure was performed under general anesthesia; a single transseptal puncture was done to access the left atrium (LA). Anticoagulation was maintained with intravenous heparin, with a targeted activated clotting time of 300–350 s, while the patient was on uninterrupted apixaban. The LA was mapped using an OctaRay multielectrode mapping catheter (CARTO3, Biosense Webster); this map was then merged with the CTLA segmentation. This allowed us to project the course of the esophagus onto the EAM (Fig. 1A, right image). The distance, as measured on the CTLA, between the esophagus and the posterior wall of LA posterior wall was just 2.1 mm. After mapping, a 31-mm FaraWave catheter (FaraPulse, Boston Scientific) was advanced to the LA.

The two superior pulmonary veins were isolated in the usual fashion with 8 applications in total per vein, 4 applications in both basket and flower mode with appropriate rotation between pairs of applications, 5 pulses at 2000 V, and 2.5 s per application. Next, the left and right branches of the CIPT were targeted using the same 8 applications approach on each side (Fig. 1B). Finally, the inferior and superior aspect of the posterior wall was targeted using the flower shape. The electroanatomical map was particularly useful in guiding the positioning of the ablation catheter and documenting the location of each application (Fig. 1A, right image) where the PFA catheter appears as a brown Lasso-like circular mapping catheter. A quadripolar electrode was placed to the right ventricle to allow pacing during ablation, which we routinely carry out as transient AV block can occur during pulsed field ablation (PFA). No temperature probe was used. After checking for exit block with the ablation catheter, the LA was remapped to prove that the pulmonary veins and posterior wall were isolated (Fig. 1A, right image). Total procedure time was 50 min, with a fluoroscopy dose of 45 mGy.

No procedural or periprocedural complications occurred, and the patient was discharged the following day. Our institutional protocol does not include performing gastroscopy routinely in patients after ablation for atrial fibrillation. High-dose esomeprazole was prescribed for 6 weeks post procedure. After 4 months of initial follow-up, sinus rhythm was maintained and the patient remained symptom free.

CIPT is a rare variant affecting less than 1% of patients undergoing ablation for atrial fibrillation [1, 2]. In such patients, both inferior pulmonary veins drain via a common trunk. Consequently, the esophagus is in contact inferiorly, posteriorly, and superiorly with CIPT. Hence, ablation with thermal energy exposes such patients to an increased risk of atrioesophageal fistula [1]. Because of the higher risk of fistula, some authors have elected to not isolate the CIPT when found during a radiofrequency pulmonary vein isolation procedure [2]. This highlights the importance of pre-procedure computed tomography of the left atrium in recognizing this variant [3, 4].

We hypothesize that PFA which allows for selective ablation of atrial myocytes offers a means of significantly safer ablation in these patients in contrast to thermal ablation modalities. It has already been shown that posterior wall isolation can be successfully performed by PFA, even in a convergent hybrid endo-epicardial approach [5]. Here, we demonstrate an effective ablation strategy combining 3D- electroanatomical mapping and PFA, which can be considered in challenging cases like CIPT.
